# Diagnostic yield of repeat sampling with immunoassay, real-time PCR, and toxigenic culture for the detection of toxigenic *Clostridium difficile* in an epidemic and a non-epidemic setting

**DOI:** 10.1007/s10096-015-2484-9

**Published:** 2015-09-16

**Authors:** J. van Prehn, C. M. J. E. Vandenbroucke-Grauls, Y. H. van Beurden, R. van Houdt, S. Vainio, C. W. Ang

**Affiliations:** Department of Medical Microbiology and Infection Control PK 1 X 124, VU University Medical Center, De Boelelaan 1118, 1081 HV Amsterdam, The Netherlands

## Abstract

Current international guidelines lack definite conclusions regarding repeat stool sampling for the detection of toxigenic *Clostridium difficile*. We assessed the value of repeat sampling and compared the diagnostic yield in an epidemic to a non-epidemic setting. Consecutive fecal samples obtained during two time frames were analyzed using direct stool immunoassay toxin testing (enzyme immunoassay [EIA]), direct stool real-time PCR toxin gene testing, and toxigenic culture. Samples collected within 7 days of the initial sample were considered repeat tests. In the epidemic setting 989 patients were analyzed, and in the non-epidemic setting 1,015. In the epidemic setting 204 patients had two or more specimens included for analysis and in the non-epidemic setting 287 patients. In the epidemic setting 136 samples yielded a positive results, either by EIA or toxigenic culture; of these, 108 were positive according to EIA and 123 according to toxigenic culture. In the first test round 98 (90.7 %, 95 % CI 85.3 to 96.2), 114 (92.7 %, 88.1 to 97.3), and 126 (92.6 %, 88.3 to 97.0) positives were detected. Subsequent test rounds yielded 10 (9.3 %, 3.8 to 14.7), 9 (7.3 %, 2.7 to 11.9), and 10 (7.4 %, 3.0 to 11.7) extra positives. In the non-epidemic setting EIA, toxigenic culture and PCR detected 33, 66, and 83 positives. The three tests combined 93 detected positives. In the first test round 30 (90.9 %, 81.1 to 100.7), 63 (95.5 %, 90.4 to 110.5), 76 (91.6 %, 85.6 to 97.5), and 87 (93.5 %, 88.6 to 98.5) positives were detected. Subsequent test rounds yielded 3 (9.1 %, −0.7 to 18.9), 3 (4.5 %, −0.5 to 9.6), 7 (8.4 %, 2.5 to 14.4), and 6 (6.5 %, 1.5 to 11.4) extra positives. In conclusion, repeat testing resulted in 4.5 % to 9.3 % extra positives. No significant difference between the settings studied could be demonstrated. Repeat sampling and multimodality testing may be chosen in an outbreak situation to detect all cases, effectively controlling nosocomial spread.

## Introduction

Rapid diagnosis of *Clostridium difficile*-associated diarrhea (CDAD) is important for both therapeutic purposes and the timely application of adequate infection control measures. Several diagnostic tests for the detection of toxigenic *C. difficile* are available. Bacterial culture is an important modality as it yields isolates for (ribo-)typing, but it is hampered by the relatively long time interval before reporting results. Immunoassays are directed to either an enzyme carried by *C. difficile* (glutamate dehydrogenase) or toxins produced by *C. difficile* (TcdA and TcdB). These immunoassays are more rapid diagnostic tests, but it is well known that direct immunoassay testing of stool samples lacks adequate sensitivity for the detection of toxigenic *C. difficile*, with a sensitivity of 60–70 % [1–3]. Real-time polymerase chain reaction (PCR)-based diagnosis has proved to be highly sensitive and has become more widely available [1, 4].

Several multiple-step algorithms, with combinations of different diagnostic tests, have been proposed to achieve acceptable turn-around times and adequate sensitivity [2, 5–11]. Routinely ordering repeat testing for *C. difficile* is generally discouraged in a non-epidemic setting as it does not seem to increase diagnostic yield sufficiently [10–12]. It may also lead to more false-positive results as has been pointed out by a modelling study that assumes that test performance does not change during repeat testing [13]. The discouragement of routine repeat testing may lead to the false assumption that there is no value in repeat sampling at all. However, repeat testing seems beneficial in outbreak situations in which the prevalence is higher [14]. This has been underlined by the 2014 ESCMID guidelines, although the level of recommendation was graded only 3 out of 4 [11]. In the 2010 IDSA guidelines “the role of repeated stool during the same episode of illness” is defined as a research gap. In the present study, our primary aim is to assess the value of repeat sampling for several widely used test modalities. Our secondary aim is to compare the diagnostic yield of repeat testing during a *C. difficile* hospital epidemic and compare this with a non-epidemic setting.

## Materials and methods

### Specimens

The results of stool testing for *C. difficile* during an epidemic and non-epidemic setting at our university hospital were analyzed. The results were prospectively entered into a database, which was retrospectively queried. Consecutive samples taken from January through December 2013 were included for the analysis of the epidemic setting. Consecutive samples taken from April 2014 through March 2015 were included for the non-epidemic analysis. Diagnostic tests were performed according to our institutional protocol: stool samples were tested directly with an immunoassay for the presence of toxins A and B, and were tested using toxigenic culture. All stool samples from the non-epidemic timeframe and a consecutive subset from the epidemic timeframe (from the end of November 2013 onward) were also tested directly with real time-PCR for toxin A and B genes. Tests that did not include all available modalities as described above were excluded from the analysis.

### Direct toxin testing

Stool samples were tested for toxins A and B using the VIDAS CDAB enzyme-linked fluorescence assay (Biomérieux). Standardized samples of stool (200 μl) were added to 1 ml of diluent and centrifuged for 5 min at 12,300 rpm. Subsequently, 300 μl of supernatant was added to the sample well of the CDAB kit. Based on the fluorescence, results were reported as positive (test value ≥ 0.37), equivocal (≥0.13 to 0.37), and negative (<0.13).

### Toxigenic culture for *C. difficile*

Stool samples were suspended in 95 % ethanol and incubated for 1 h at room temperature. A sample of the suspension was then inoculated on selective CLO agar (cycloserine 100 μg/ml, cefoxitin 8 μg/ml, and amphotericin B 2 μg/ml; Biomérieux) and incubated for 3 days at 37 °C under anaerobic conditions. Suspect colonies were confirmed to be *C. difficile* by matrix-assisted laser desorption/ionization time-of-flight mass spectrometry with the Vitek MS system (Biomérieux). In the epidemic setting positive toxigenic culture was defined as a positive direct stool toxin testing + cultured *C. difficile* or a cultured *C. difficile* isolate with positive toxin testing of the isolate. To this end, isolates were inoculated in a chopped-meat glucose broth [15]. After 1 and 2 days of incubation a sample of the broth was tested with the VIDAS CDAB immunoassay for the presence of toxins A and B. In the non-epidemic setting positive toxigenic culture was defined as a positive direct stool toxin gene assay + cultured *C. difficile* or a cultured *C. difficile* isolate with subsequent positive toxin gene testing.

### Real-time PCR

A standardized amount of stool was dissolved in 1 ml S.T.A.R.-buffer (Roche Diagnostics) and kept at −80 °C for at least one hour. After 10 min at 100 °C, DNA was extracted using the MagNA-Pure96 platform (Roche Diagnostics). DNA was then amplified using a Real-Time PCR targeting the *C. difficile* toxin genes *cdtA* and *cdtB*, as previously described by de Boer et al. [16]. This PCR was performed using the LightCycler480 platform and software (Roche Diagnostics).

### Analysis

All samples from a patient collected within 7 days of the first sample were considered to be repeat tests. Test results of samples collected after this 7-day period were not included in the patient level analysis. All patients were clinically suspected of CDAD and when a positive result was reported for either test modality, they were treated accordingly (cessation of antibiotic therapy where possible and/or metronidazole or vancomycin per os) and appropriate infection prevention measurements were taken. The diagnostic yield of extra test rounds was calculated using 95 % confidence intervals (asymptotic Wald method).

## Results

### Sample level

**Fig. 1:**
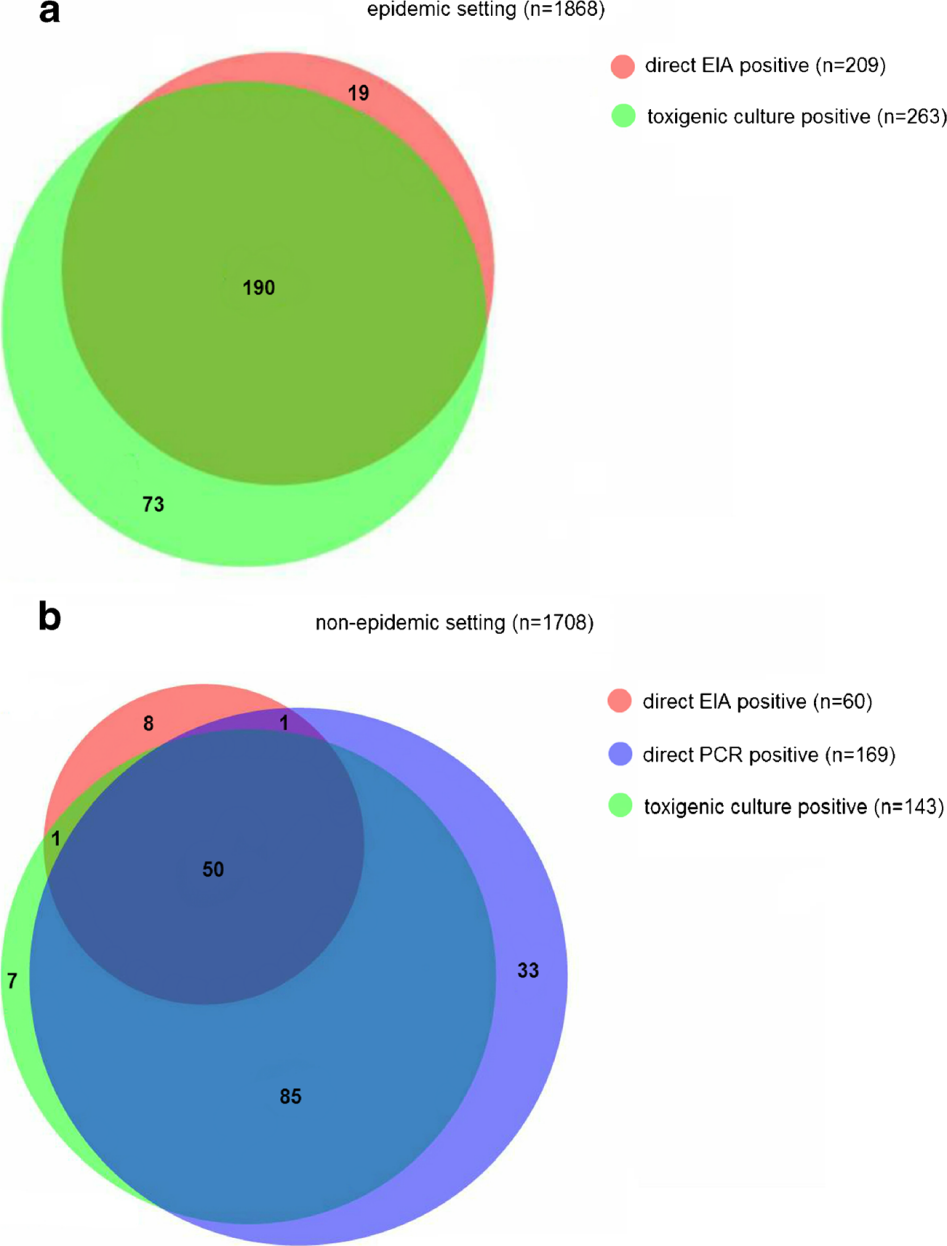
Venn diagram of sample level analysis in** a** the epidemic timeframe and** b** the non-epidemic timeframe. Samples with a positive result are shown. In the epidemic timeframe there were 1,586 samples with both a negative direct enzyme immunoassay (EIA) and toxigenic culture. In the non-epidemic timeframe there were 1,523 samples with a negative direct EIA, negative direct polymerase chain reaction (PCR), and a negative toxigenic culture (8 PCR results that were not interpretable were regarded as negative)

During the epidemic setting, 1,883 stool samples were taken, of which 11 were excluded because no culture result was available, and 4 because no direct immunoassay was available. After exclusion, there were 1,868 samples available for analysis of the epidemic timeframe. During the non-epidemic setting 1,745 stool samples were taken, of which 37 were excluded: 3 had no direct PCR and direct immunoassay on stool available, 16 had no culture, 13 had no direct PCR on stool, and 5 had no direct immunoassay test. After exclusion, there were 1,708 samples available for analysis of the non-epidemic timeframe. The results of this analysis and the overlap of the positive test results are shown in a Venn diagram in Fig. 1a for the epidemic timeframe, and in Fig. 1b for the non-epidemic timeframe [17]. In the epidemic timeframe there were 282 samples that tested positive with either immunoassay or toxigenic culture, 263 of which were toxigenic culture positive; in 19 samples toxin was demonstrated while *C. difficile* was not cultured. In the non-epidemic timeframe there were 185 samples that tested positive with either immunoassay, toxigenic culture, or PCR; 60 of these samples tested positive with immunoassay, 169 with PCR, and 143 with toxigenic culture. In 9 samples toxin was demonstrated using immunoassay, while *C. difficile* was not cultured. There were 33 stool samples in which toxin genes were demonstrated while no toxin was detected and no *C. difficile* was cultured.

### Patients

In the epidemic setting, 989 patients were analyzed, and in the non-epidemic setting 1,015 patients were analyzed. The number of patients that were included per test round for each timeframe can be found in Table 1. In the epidemic situation, 136 patients tested positive with either toxin testing or toxigenic culture. In the non-epidemic setting a total of 93 patients tested positive with either toxin testing, toxigenic culture or toxin gene testing. The diagnostic yield of repeat testing in the epidemic situation ranged from 7.3 to 9.3 %, for detailed results see Table 2. Subset analysis of 137 patients tested with PCR, 30 of whom tested positive, indicated a diagnostic yield of 10 % of repeat PCR testing in the epidemic situation (Table 3). Repeat testing in the non-epidemic situation resulted in a diagnostic yield ranging from 4.5 % to 9.1 %, for detailed results see Table 4. The 95 % confidence intervals of the diagnostic yield of repeat testing showed overlapping results, indicating no significant difference between the epidemic and non-epidemic setting.

**Table 1:** Number of patients included per test round for the epidemic and non-epidemic timeframe

	1st test *n*	2nd test *n*	3rd test *n*	4th test *n*	5th test *n*	6th test *n*
Epidemic timeframe	989	204	35	5	2	0
Non-epidemic timeframe	1,015	287	55	8	0	0

Tests were only considered to be repeat tests when samples of a patient were collected within 7 days of the first sample

**Table 2:** Diagnostic yield of repeat testing in the epidemic setting

			1st test		2nd test		3rd test		4th test	5th test
Test modality	*n*	*n*_positives total_	*n*_positives_	% (95 % CI)	*n*_positives_	% (95 % CI)	*n*_positives_	% (95 % CI)	*n*_positives_	*n*_positives_
EIA	989	108	98	90.7 (85.3 to 96.2)	10	9.3 (3.8 to 14.7)	0	0 (0 to 0)	0	0
Toxigenic culture	989	123	114	92.7 (88.1 to 97.3)	8	6.5 (2.1 to 10.9)	1	0.8 (−0.8 to 2.4)	0	0
Any of the two modalities	989	136	126	92.6 (88.3 to 97.0)	9	6.6 (2.4 to 10.8)	1	0.7 (−0.7 to 2.2)	0	0

The number of positive patients detected by the different diagnostic modalities is shown with the percentage diagnostic yield per test round and 95 % confidence intervals

*EIA* enzyme immunoassay

**Table 3:** Diagnostic yield of repeat polymerase chain reaction (PCR) in the epidemic setting

			1st test		2nd test		3rd test		4th test
Test modality	*n*	*n*_positives total_	*n*_positives_	% (95 % CI)	*n*_positives_	% (95 % CI)	*n*_positives_	% (95 % CI)	*n*_positives_
PCR	137	30	27	90.0 (79.3 to 100.7)	3	10.0 (−0.7 to 20.7)	0	0 (0 to 0)	0

Analysis of a consecutive subset tested with PCR. The number of positive patients detected is shown with the percentage diagnostic yield per test round and 95 % confidence intervals

**Table 4:** Diagnostic yield of repeat testing in the non-epidemic setting

			1st test		2nd test		3rd test		4th test
Test modality	*n*	*n*_positives total_	*n*_positives_	% (95 % CI)	*n*_positives_	% (95 % CI)	*n*_positives_	% (95 % CI)	*n*_positives_
EIA	1,015	33	30	90.9 (81.1 to 100.7)	3	9.1 (−0.7 to 18.9)	0	0 (0 to 0)	0
PCR	1,015	66	63	95.5 (90.4 to 100.5)	3	4.5 (−0.5 to 9.6)	0	0 (0 to 0)	0
Toxigenic culture	1,015	83	76	91.6 (85.6 to 97.5)	6	7.2 (1.7 to 12.8)	1	1.2 (−1.1 to 3.6)	0
Any of the three modalities	1,015	93	87	93.5 (88.6 to 98.5)	5	5.4 (0.8 to 10)	1	1.1 (−1.0 to 3.2)	0

The number of positive patients detected by the different diagnostic modalities is shown with the percentage diagnostic yield per test round and 95 % confidence intervals

## Discussion

In the present study we evaluated repeat *C. difficile* testing using several diagnostic modalities and found that repeat sampling resulted in 4.5 % to 9.3 % extra positives. We could not demonstrate a significant difference between the epidemic and non-epidemic settings studied. The largest increase in positive patients was found when we used all tests on the primary sample, compared with direct PCR only. Direct PCR testing with repeat samples yielded a few more positive patients. The same was true, to a lesser extent, when toxigenic culture was added to direct PCR. The highest number of positive patients will be detected with multiple modality testing and repeat testing.

To prevent bias caused by patient-to-patient transmission, we have limited the interval of repeat testing and only analyzed the first episode of test series per patient. As the Venn diagrams of the sample level analysis clearly illustrate, it is hard to define a true gold standard when all modalities used seem to miss some positives that are detected using other modalities; however, Fig. 1b illustrates that PCR testing will result in the highest detection rate of patients with a positive immunoassay, PCR or toxigenic culture. In clinical practice we initiated CDAD treatment and infection prevention measures if any of the tests were positive. It is of interest that of the 33 samples that only tested positive with PCR, several samples belonged to patients who tested positive with other modalities before or afterward, i.e., patients with emerging or regressing disease. This probably indicates that the PCR is indeed more sensitive than the other tests studied. In the present study, we have regarded equivocal EIA results as negative. Of the 63 samples with equivocal results in the non-epidemic setting, 47 were negative with both direct PCR and toxigenic culture, and 16 were positive with both direct PCR and toxigenic culture. If these equivocal results were regarded as positive, this would lead to an increase in concordance between EIA and PCR/toxigenic culture. However, this would also lead to an even larger increase in samples that were positive only according to EIA; these could be false-positives.

Our study has some limitations. In the epidemic setting, only a consecutive subset of the patients received a PCR test; however, this subset contains a considerable number of patients with a positive test. Furthermore, in the non-epidemic setting, PCR analysis was carried out on all samples included in the study (and no significant differences in diagnostic yield of repeat testing with any modality was demonstrated between the two settings). It could be argued that a multicenter study would be necessary to externally validate our results. However, our results are based on a commonly used and commercially available immunoassay, standardized regular bacterial culture techniques, and a previously described PCR method.

Our results agree with the outcome of the study by Debast et al., who found that repeat *C. difficile* toxin testing of stools within 1 week yielded 5 % of positive patients [14]. They concluded that repeat toxin testing of stools is of value for controlling outbreaks of *C. difficile* infection. From our own experience, we agree with this point of view. Other studies found a lower diagnostic yield from repeat testing [12, 18]. For example, in a study by Aichinger et al. it was concluded that repeat testing by enzyme immunoassay and PCR is of little value, as diagnostic gains of less than 2 % were calculated [12]. A more recent study by Green et al. found that only 11 out of 1,066 PCR tests repeated within 7 days (1 %) were positive [18]. When taking into account the costs of routinely ordering repeat testing and the low diagnostic yield, it seems inadvisable to routinely apply repeat testing in the non-epidemic setting [19].

As pointed out in a paper by Peterson and Robicsek, there is a risk of more false-positives with repeat sampling, assuming that test performance does not change with repeat sampling [13]. However, in clinical practice repeat testing is ordered, especially in patients with continuing clinical suspicion of CDAD, thereby altering the a priori chance of a positive test result, which affects test characteristics and subsequently diagnostic yield. Although we could not demonstrate a statistically significant difference in the diagnostic yield of repeat testing between the two settings, the absolute diagnostic yield in the epidemic setting will be higher by definition. Furthermore, in an outbreak situation it is paramount to have the highest sensitivity possible and to find every CDAD case. To this end, it might be better to accept more false-positives and apply repeat sampling and multimodality testing to increase sensitivity and effectively control nosocomial spread.
